# Using crossmodal correspondences as a tool in wine communication

**DOI:** 10.3389/fpsyg.2023.1190364

**Published:** 2023-07-31

**Authors:** Anders Crichton-Fock, Charles Spence, Nicklas Pettersson

**Affiliations:** ^1^School of Hospitality, Culinary Arts & Meal Science, Örebro University, Örebro, Sweden; ^2^Crossmodal Research Laboratory, Department of Experimental Psychology, Oxford University, Oxford, United Kingdom; ^3^Örebro University School of Business, Örebro, Sweden

**Keywords:** crossmodal correspondence, multisensory experience, crossmodal communication, product matching, wine communication

## Abstract

**Introduction:**

This research investigates consumer acceptance of alternative methods for communicating information about wine, focusing on the alignment between sensory attributes and consumer expectations.

**Methods:**

A survey was administered to wine enthusiasts to assess their attitudes toward crossmodal communication.

**Results:**

The findings reveal significant associations between consumer behaviors and acceptance of alternative communication methods, highlighting the emerging field of crossmodal correspondences.

**Discussion:**

These results suggest that leveraging crossmodal correspondences can enhance the match between a product’s sensory qualities and consumer expectations, potentially reducing wine wastage resulting from unmet consumer preferences. These findings have implications for improving communication strategies in the wine industry and enhancing consumer experiences.

## 1. Introduction

Multisensory research is important if one is to understand how to optimize communication, since it involves much more than just spoken or written language. As humans, we use multiple senses such as sight, hearing, touch, taste, and smell, to gather information and communicate with others. Research on the crossmodal correspondences can provide an efficient tool for communication by leveraging the connections between the senses, not least when it comes to wine (see Spence, 2023). This is accomplished by involving a combination of different senses to increase the potential success of product communication, depending on the product, potential consumer, and purpose. For example, this could include different kinds of visual cues, such as shapes and colors (Spence, 2023), to support the communication of complex multisensory products, which on a single-sense basis can be hard to fully communicate/understand for the regular consumer. It could also be the use of mental pictures, metaphors and analogies relating to common recognizable human characteristics (Herdenstam et al., 2009, 2018, 2020). In this context it could be argued that consumers in general are well-experienced using crossmodal descriptions and sensory metaphors in their daily speech. For example: *warm welcoming* and *bubbly personality* (touch), *bright idea* and *glowing review* (vision), *end on sour note* and *such a sweet personality* (taste), *love stinks* and *sweet smell of success* (odor), *music to my ears* and *the world is listening* (sound).

In this context, a question arises as to whether this could be a tool not only to help understand the correspondence between different senses, but also as a tool to meet future challenges in regard to food communication and ultimately food waste (Uiterkamp and Vlek, 2007; Rosen, 2009; Adams, 2010; Hopton et al., 2010; Stock and Burton, 2011; De Soete, 2016; Rinaldi, 2017; Dondi et al., 2020; Martini et al., 2021). As mentioned already, there is a need to implement new strategies to decrease the climate footprint, both in the wine industry as well as beyond (Christ and Burritt, 2013; Galbreath et al., 2020). Strong communication and learning from the sensory perspective of crossmodal correspondence can potentially be used to enhance sensory training by creating more immersive and interactive learning experiences as a tool to help individuals better retain information about a product (Ahn, 2011; Ghosh et al., 2016), especially one with a complex flavor profile such as wine. This is a critical subject when it comes to the communication of olfactory experiences, both with regard to limitations in verbally and linguistically grasping the message as well as in terms of understanding sensory complex food products such as wine (Paradis and Eeg-Olofsson, 2013).

In the realm of sensory marketing, the utilization of multisensory stimulation has traditionally served as a valuable tool for understanding consumer responses to products in relation to the fulfillment of their expectations (Elder and Krishna, 2010; Varela and Ares, 2012; Spence et al., 2013; Krishna and Schwarz, 2014; Croijmans and Wang, 2021; Spence, 2022a). This line of research focuses, in part, on consumer acceptance while acknowledging the efficacy of imagery and text in marketing, as well as the interaction between visual and linguistic elements in such contexts (Bolognesi and Strik Lievers, 2018). Nonetheless, it is crucial to explore how various visual cues (e.g., Lick et al., 2017; Baptista et al., 2022; Nguyen and Durner, 2023; Spence, 2023) and haptic sensations (Piqueras-Fiszman and Spence, 2012; Wang and Spence, 2018) may influence consumer preferences and correlate with taste and texture. Within this context, research has indicated that imagery depicting wine plays a significant role in the recollection and communication of sensory experiences (Herdenstam et al., 2009, 2018; Croijmans et al., 2020) and that certain training and expertise can enhance imagery abilities among wine-interested consumers (Croijmans et al., 2020; Herdenstam et al., 2020). In a recent study, consumers with varying levels of self-reported imagery vividness were examined (Croijmans and Wang, 2021). The findings suggested that the vividness of mental imagery might be a crucial factor to consider, as multisensory wine descriptions can help stimulate purchase intentions among consumers with lower imagery vividness, particularly in terms of their desire to drink the wine (Croijmans and Wang, 2021). Conversely, consumers with higher vividness reported a greater desire to drink the wine even in the absence of multisensory descriptions in imagery (Wang et al., 2022).

Overall, the understanding of mental imagery vividness, is an important factor to consider when it comes to finding better tools for the communication of wine (Spence, 2017; Spence, 2022c). This approach might be applied for understanding how other complex food products communicate, as they contain layers of volatile odors, flavors, and tactile sensations, and this tool might be critical in understanding linguistic communication and different consumer group’s attraction to a product (Paradis and Eeg-Olofsson, 2013). In this context, investigating the multisensory environment and its impact on consumer acceptance has also shown how important it is to consider the sound and hearing aspects when tasting wine (Spence and Wang, 2015a,b,c).

Recent research studies have shifted their focus towards comprehending the comprehensive impact of multisensory environments, surpassing earlier studies that examined sensory experiences on an individual basis. These studies aim to gain insights into consumer experiences (Maziriri et al., 2021; Spence and Van Doorn, 2022; Wörfel et al., 2022; Spence, 2022a). This emphasis is particularly significant in unraveling the reasons behind consumers’ product choices (Spence, 2020, 2022a,b).

In the past, a large body of wine research has demonstrated a wide variety of influences affecting the consumer’s esthetic and hedonic relation to sensory experience (Spence et al., 2014). One possible reason is that traditional and cultural aspects of the wine industry are reflected in idyllic images with beautiful landscapes, which has led to a perception that wine is considered as an environmentally friendly product (Ruggieri et al., 2009; Christ and Burritt, 2013). On the contrary, the United Nations has made it clear that there is an inevitable need to increase resources used in the food industry through environmental, sustainable, and cost-effective solutions (Roy et al., 2009; Ghosh et al., 2016; Merli et al., 2018). According to the above-mentioned studies, it can be argued that finding alternative ways to communicate about wine and other complex food products, novel or otherwise, could be helpful to both the consumer experience and the environment (Ahn, 2011; Ghosh et al., 2016). This is partially due to the demonstrated limits of language in describing sensory experiences that derive from the olfactory domain when experiencing wine (Paradis and Eeg-Olofsson, 2013).

This questionnaire-based study aimed to explore the attitudes of wine consumers towards crossmodal communication and assess the effectiveness of crossmodal correspondence as a potential communication tool for wine and other complex food products. The primary motivation behind this study was to explore innovative approaches to enhance the alignment between consumer expectations and sensory experiences, particularly by identifying novel and effective tools to address challenges associated with food production and waste.

## 2. Materials and methods

### 2.1. Ethics statement

All of the participants were over 20 years of age, and informed consent was obtained from all of those taking part in the study. All of the data and analysis files were kept in accordance with legislated and regulated data handling practices.

### 2.2. Participants

The participants consisted of 329 students from different sections of the 7.5-credit, 15-week distance course ‘Beverage knowledge’ offered through Örebro University (Sweden). The students received training in which they learned a common methodology to analyze wine. One important factor in the selection of these group of participants was their common experience of partly being exposed of effects of crossmodal correspondence during their training sessions, learning the basics of wine tasting. Both theoretically, by taking part of research during the course, and also practically, through the tasting exercises, experiencing crossmodal correspondences affects when moving from one sense modality to another. They were instructed first to look at a wine’s appearance qualities in order to make visual judgments concerning its color, intensity, maturity, age, freshness, acidity, and concentration. They were then encouraged to validate these first impressions on the nose, and subsequently on the palate, observing the effect of crossmodal correspondence and their notable impact during the professional wine tasting procedures, assessing first visual impressions, then the bouquet with layers of aromas, and lastly taste and oral-somatosensation while during this multisensory experience also being intervened by the retronasal effect of the odors in the palate. The majority of the participants were female (59%) and lived in the city (76%). Almost all had previously studied at university (94%), and most of them had received a bachelor’s degree, or higher (74%). As a group, they considered that they had better than average wine knowledge (76%). Most of the participants consumed wine on a weekly basis (87%), which they typically purchased at Systembolaget (Sweden’s nationally regulated liquor monopoly; 81%) and then consumed at home (80%; see Appendix A).

The participants shared the following traits:

- They had all tasted the same wines and other beverages and had therefore shared a variety of sensory experiences within their training program.
- They had all learned a common approach and methodology when it came to the sensory analysis of wine. It can therefore be presumed that, as a group, they had an awareness of the importance of (all) the senses in the analysis process, including vision, olfaction, taste, touch, and sound.
- They had all experienced crossmodal correspondence during the tasting methodology practice. In other words, they were aware that the senses could not be totally isolated from one other during the process of analyzing the wine and, subsequently, communicating about the multisensory experience.

### 2.3. The questionnaire

The questionnaire used in this study comprised four sections. This section focuses on the analysis of the first section, which included inquiries about demographics, as well as single-choice and multiple-choice (check-all-that-apply; CATA) questions pertaining to purchase and consumption behaviors, communication practices, and sensory experiences related to wine. The remaining sections, which investigated visual aspects, the use of specific symbols, and alternative communication methods, were analyzed and presented separately based on the results of the statistical analysis. The questionnaire was distributed to participants upon completion of the course. In addition to collecting demographic information, it encompassed single-choice and multi-choice (CATA) questions concerning purchase behaviors, consumption habits, communication preferences, and sensory experiences (see Appendix B).

#### 2.3.1. Examples of single-choice questions

1) Where do you primarily purchase wine? 2) Where do you primarily consume wine? 3) To what extent would you consider buying a wine based only on sensory information – i.e., if no other conventional information were available?

#### 2.3.2. Examples multi-choice questions (CATA)

1) When purchasing wine from your selected choice in the question above, what factors influence your choice? 2) When consuming your wine at your selected choice in the question above, which factors primarily influence “your experience”? 3) If you could freely choose an alternative to regular communication on a wine label, what would you prefer?

### 2.4. Data analysis

EyeQuestion version 5 (Logic 8, Elst, Netherlands), a software program for sensory and consumer testing, was used to collect the participants’ responses. The software package R (R Core Team, 2021) was used to analyze the data.

## 3. Results

We analyze the influential factors when purchasing and consuming wine (in section 3.1) and reported preferences towards alternative wine communication (in section 3.2) including their associations to demographics and influential factors (in section 3.2.1–7). The relations between preferences are then analyzed (in section 3.3) and followed up by investigating the most frequent preference combinations and their associations to demographics and influential factors (in section 3.4).

### 3.1. Reported factors influencing purchasing and consumption of wine

The most influential factors reported by the participants when purchasing wine were (in descending order of importance): type of grape (77%), prior experience of the wine (72%), country of origin (72%), external recommendations (68%), style (65%), and price (62%). At the same time, the sensory descriptors communicated on the wine label or on the shelf were also reported to be an important factor for just under half of the participants.

The dominant factors influencing the choices reported by this group of engaged wine consumers would appear to be of a more general character. On the other hand, more specific aspects such as: sensory indicators of the experience, name of the producer, alcohol or vintage information, overall product communication in the store, shelf information and producer information (on the bottle design) were considered to be less important (see Table 1).

**Table 1:** Reported factors influencing wine purchasing behavior (CATA).

Influence factor	*n*	Frequency % (*n* = 329)
The grape	253	76.90%
Previous experience (having tried the wine before)	237	72.04%
Country of origin	237	72.04%
External recommendations (professional, friends, others)	224	68.09%
Style of wine	215	65.35%
Price	204	62.01%
Sensory indicators (concerning the taste, aroma profile, and/or mouthfeel)	163	49.54%
The wine producer	112	34.04%
Climate impact (How eco-friendly is the wine)	98	29.79%
Vintage	65	19.76%
Illustrations on the label	61	18.54%
The front label	61	18.54%
Bottle design (bag in box/bottle)	55	16.72%
The back label	12	3.65%
Other	9	2.74%

The most influential sensory factors reported by the group of wine enthusiasts when consuming wine were as follows: taste (94%), tasting/dinner setting (66%), and smell of the wine (65%). Far fewer of the participants reported being influenced by the other senses during their consumption: vision (26%), touch (12%), sound (8%). Here, a discrepancy was noted, with the reported factors influencing purchasing being dominated by more general information rather than sensory indicators, and the sensory indicator of taste was reported as the single most important factor when consuming the wine (see Table 2).

**Table 2:** Reported factors influencing experience of consuming wine (CATA).

Influence factor	*n*	Frequency % (*n* = 329)
Taste	309	93.92%
Dinner/tasting setting	216	65.65%
Smell/odor sensations of the wine	215	65.35%
“Meal” companions/other guests	164	49.85%
The visual impression (vision)	84	25.53%
Overall room environment	75	22.80%
Tactile (touch) sensations	40	12.16%
Hosts/Professionals/Staff	31	9.42%
Sound environment (sounds)	25	7.60%
Others	3	0.91%

### 3.2. Reported attitudes towards alternative wine communication

When the participants were asked whether they might consider buying wine based only on sensory information and no conventional label information, most of them had positive reactions to buying a bottle just so long as the sensory descriptors matched their own personal preferences (91%). Another question asked if they would also consider buying a wine with no specific origin, for example, a blend of different wines from different origins. Here, the participants reacted positively to the thought of buying a bottle if the descriptors happened to be equivalent to their sensory profile (95%). In terms of sustainability, 76% of them answered that they, to varying extents, would consider climate change into when making a decision about what wine to buy (see Appendix C).

In the questionnaire, the participants were introduced to alternative ways of communicating about the sensory experiences of wine and were asked to freely choose from examples of different modalities (see Appendix B). Visual communication using shapes and colors were the most frequently requested (54%), while flavor (45%) and odor/aroma (38%) were also highly ranked alternative means of communication. The least popular alternatives were those communicated auditorily, such as speech (9%), music (8%), and sounds (6%), see Table 3.

**Table 3:** Reported alternative communication to regular wine label (CATA).

[Article section] Alternative communication	*n*	Frequency % (*n* = 329)
[3.2.1] Shapes/Colors (other visual symbols)	178	54.10%
[3.2.2] Flavors (tastes)	148	44.98%
[3.2.3] Odors/aromas (smells)	125	37.99%
[3.2.4] Touch (tactile)	49	14.89%
[3.2.5] Speech (hearing)	30	9.12%
[3.2.6] Music (hearing)	26	7.90%
[3.2.7] Sounds (hearing)	20	6.08%

In sections 3.2.1–3.2.7 we explore each of the alternative ways of communicating in relation to reported demographics (see also Appendix D) and purchase choice (Appendix E) and consumption experience (Appendix F), using two-sided hypothesis tests of, association (Kendall’s Tau rank correlation coefficient, Fisher’s exact test and Chi-square test), and of equal location among groups (Mann–Whitney U test and for significance to categories Kruskall-Wallis test, Wilcoxon rank sum test and t-test), see Appendix D. When studying the relation to purchase choice and consumption experience, as to rule out the influence of the reported locale, we also use binary logistic regression to regress the preferred alternative way of communication on the locale and the influential factor under study. Logged odds ratios (logOR) are used to represent 2*2 categorical associations, as exemplified at the start of section 3.2.1.

#### 3.2.1. Shapes/colors (other visual symbols)

There were 62 males and 115 females who preferred shapes/colors as a means of communication, while 115 males and 80 females did not. A positive (negative) logged odds ratio indicates larger (smaller) odds amongst males who preferred shapes/colors as communication, relative to the odds among females, while a value of zero indicates no difference in odds. Since the odds amongst females (115/80 = 1.44) is higher than amongst males (62/70 = 0.89), the positive logged odds ratio, logOR = log(1.44/0.89) = 0.48, indicates that females were more likely than males to prefer shapes/colors as a means of communicating about wine. Using Fisher’s exact test, under the null hypothesis that gender and preference for shapes/colors are independent, the probability of an outcome at least as extreme as the observed (i.e., the *p*-value) is 0.042, which is less than 0.05. Thus, females (59%) are significantly more open to communications involving vision (using shapes/colors) to describe the multisensory experience of wine, compared to males (41%), see Tables AD.1, AD.2.

Regarding reported influencing purchasing factors (Table AE.1), the odds of preferring visual communication were significantly smaller for respondents influenced (relative to those not influenced) by vintage (logOR = −0.63), and significantly larger for respondents influenced by the climate impact (0.66), the bottle design (0.76), the illustrations on the label (1.15) or the front label (0.67). The sensory indicators (0.48) and the price (0.50) were also significantly larger, but not after controlling for the locale of purchase (Table AE.2).

Among the factors influencing the experience (Table AF.1), the odds of preferring visual communication were significantly larger for respondents influenced by taste sensations (logOR = 1.34) or hosts/professionals/staff (0.55), also after controlling for the locale of consumption (Table AF.2).

#### 3.2.2. Flavors (tastes)

Neither any demographical factors (Appendix D) nor any factors influencing wine purchasing (Appendix E) were significantly related to preferring flavors (tastes) as means of communication. The only association found to be statistically significant (Table AF.1), which also held after controlling for the locale of consumption (Table AF.2), was the smaller odds of preferring flavors (logOR = −0.55) for respondents whose experience was affected by the overall room environment.

#### 3.2.3. Odors/aromas (smells)

The median income was significantly lower amongst those respondents who were open to (35,000 SEK) vs. those who were not open to (40,000 SEK) communication through odor/aromas (Table AD.4 and Figure AD.2).

Among the factors influencing the purchase (Table AE.1), the odds of preferring communication via odors/aromas were significantly larger for respondents influenced by the country of origin (logOR = 0.74), the type of grape (0.70), the style (0.54), the sensory indicators (0.63) or the bottle design (0.72) respectively, also after controlling for the locale of purchase (Table AE.2).

Regarding influence on consumption experience (Table AF.1), only one association was found to be statistically significant, where the odds of preferring odor/aromas was (intuitively) larger for those whose experiencing was influenced by smell/odor (logOR = 0.86), also after controlling for the locale of consumption (Table AF.2).

#### 3.2.4. Touch (tactile)

Consumers open for communication involving touch were on average significantly younger (35.2 years) than those who were not open (42.4 years) to this form of communication (Table AD.3 and Figure AD.1). Their median income was also significantly lower (34.3’ SEK) compared to those who were not open to communication through touch (39’ SEK), see Table AD.4 and Figure AD.2. The respondents open to vs. not open to communication through touch differed significantly in their distribution of wine consumption, and those more open to this modality were less likely to consume wine frequently (33% more than once weekly) compared to respondents that were not open to this modality (55% more than once weekly). Also, they were significantly more likely to primarily consume wine in restaurants (12%) and not with friends or family/in a wine tasting group (8%) compared to others (4 and 15% respectively), see Table AD.5.

Regarding statistically significant associations between reported factors influencing purchase of wine (Table AE.1) and the preference for tactile alternative communication the odds was (intuitively) larger for respondents who preferred communication via touch for the illustrations on the label (logOR = 1.61) but as well for the front label (1.16), and the bottle design (0.84), also after controlling for the locale of purchase (Table AE.2).

The only association found to be statistically significant was the (intuitively) larger logged odds (logOR = 0.91) of preferring touch for consumers whose experience were influenced by tactile sensations (Table AF.1), which also held after controlling for the locale of consumption (Table AF.2).

#### 3.2.5. Speech (hearing)

Neither any demographical factors (Appendix D) nor any factors influencing the consumption (Appendix F) were significantly related to preferring speech (hearing) as a means of communication. The only association found to be statistically significant (Table AE.1) was the larger logged odds of preferring speech as alternative communication for respondents whose wine purchasing were influenced by the country of origin (logOR = 1.34), also after controlling for the locale of purchase (Table AE.2).

#### 3.2.6. Music (hearing)

Respondents open to alternative communication via music were on average significantly younger (36.4 years) compared to others (41.8), and lived in more densely populated areas (96% vs. 74% in capital or city), see Table AD.3 and Figure AD.1.

For influence on purchase and consumption of wine, only a single factor each was significantly associated to preferring music as alternative communication, also after controlling for the locale. Regarding purchase, the odds of preferring music were higher for the bottle design (logOR = 1.27), see Appendix E, and for consumption the odds of preferring music were higher for the sound environment (logOR = 1.75), see Appendix F.

#### 3.2.7. Sounds (hearing)

Respondents open to alternative communication via sounds were on average significantly younger (34.8 years) compared to others (41.7), and lived in more densely populated areas (95% vs. 74% in capital or city), see Table AD.3 and Figure AD.1.

None of the factors influencing consumption experience (Appendix F) were significantly associated with the preference for sounds as alternative communication. Regarding influence on purchase of wine a single factor was statistically significant (Table AE.1) with a larger odds of preferring sounds in relation to sensory indicators (logOR = 1.18), also after controlling for the locale of consumption (Table AE.2).

### 3.3. Associations between reported alternatives to regular wine communication

We study the pairwise associations between the seven preferences for alternative wine communication using logOR and correlations, and then look for higher dimensional structures. While most consumers reported only one alternative (45.9%), very few reported no alternative (6.1%), so almost half (49.0%) checked at least two of these alternatives (see Table 4).

**Table 4:** Total number of reported alternatives (Shapes/Colors; Sounds; Music; Speech; Touch; Odors/aromas; Flavors) to regular wine communication among respondents.

Number of reported alternatives	0	1	2	3	4	5	6	7	Total
Number of respondents	20	151	90	45	13	6	0	4	329
Percentage of total respondents (%)	6.1	45.9	27.4	13.7	4.0	1.8	0	1.2	100

When pairs of reported alternatives were considered, about half of them had a significant relationship (see Table 5). Since all significant logORs/correlations were positive, except for shapes/colors, where only touch was positive and the others significantly negative, the occurrence of a reported alternative generally had an increased probability of also having other alternatives reported. Particularly, the logOR/correlation between sounds and music were large (2.89/0.40), although both alternatives were quite infrequent in total (6.1 and 7.0%). The logOR/correlation was also fairly high between Odors/aromas and Flavor (1.32/0.31). The other logORs involving Speech, Music, Sounds and Touch (except for Music and Touch) were about as high (1.02–1.49) although the correlations were somewhat lower (0.10–0.18). For preferred alternatives Shape/colors and Flavors, the logOR was not as high (0.90) but the correlation was somewhat higher (0.22).

**Table 5:** Pairwise logged odds ratios (upper right) and correlations (lower left) of reported alternatives to regular wine communication.

	Shapes/colors	Sounds	Music	Speech	Touch	Odors/aromas	Flavors
Shapes/colors		0.26	−0.35	−0.78*	1.11***	−0.66***	−0.90***
Sounds	0.03		2.89***	1.33**	1.47***	0.74	0.64
Music	−0.05	0.40***		1.49***	0.83	0.70	0.39
Speech	−0.11*	0.14*	0.18***		1.02**	0.69	−0.22
Touch	0.18**	0.18**	0.10	0.13*		1.02***	0.09
Odors/aromas	−0.16**	0.09	0.10	0.10	0.18***		1.32***
Flavors	−0.22***	0.08	0.05	−0.03	0.02	0.31***	

Log odds ratio with Fisher’s exact test (Haldane-Anscombe), and Pearson correlation coefficient with *t*-test. Two-sided *p*-value: 0.05 > * > 0.01 > ** > 0.001 > ***.

Among the respondents there were 51 different combinations of preferences for alternative wine communication, including the combination with no preferences at all. After applying logistic principal component analysis for dimensionality reduction on the preferences, we kept the first three extracted principal components (PCs) since they accounted for as much as 79% of the total deviance among the preferences (44, 19 and 16% respectively), involved all preferences with (very) high loadings and resembled the correlational structure well, see Table 6. The first PC had high loadings (absolute value larger than 0.3) of Sounds, Music, Speech and Touch, the second had a very high loading (absolute value larger than 0.6) of Shapes/colors but also high of Odors/aromas and Flavors, while the third PC had a very high loading of Shape/colors, but also high of Odors/aromas and Touch.

**Table 6:** Loading matrix from logistic principal component analysis on preferences for alternative wine communication.

Principal component (% explained deviance)	Shapes/colors	Sounds	Music	Speech	Touch	Odors/aromas	Flavors
First PC (44%)	0.090	**−0.496**	**−0.488**	**−0.468**	**−0.464**	−0.242	−0.119
Second PC (19%)	**−0.462**	−0.092	−0.034	−0.096	−0.272	**0.449**	***0.702***
Third PC (16%)	***0.655***	−0.106	−0.180	−0.265	**0.373**	**0.537**	0.173

Loadings with absolute value larger than 0.3 (0.6) are in bold (bold italic).

**Figure 1:**
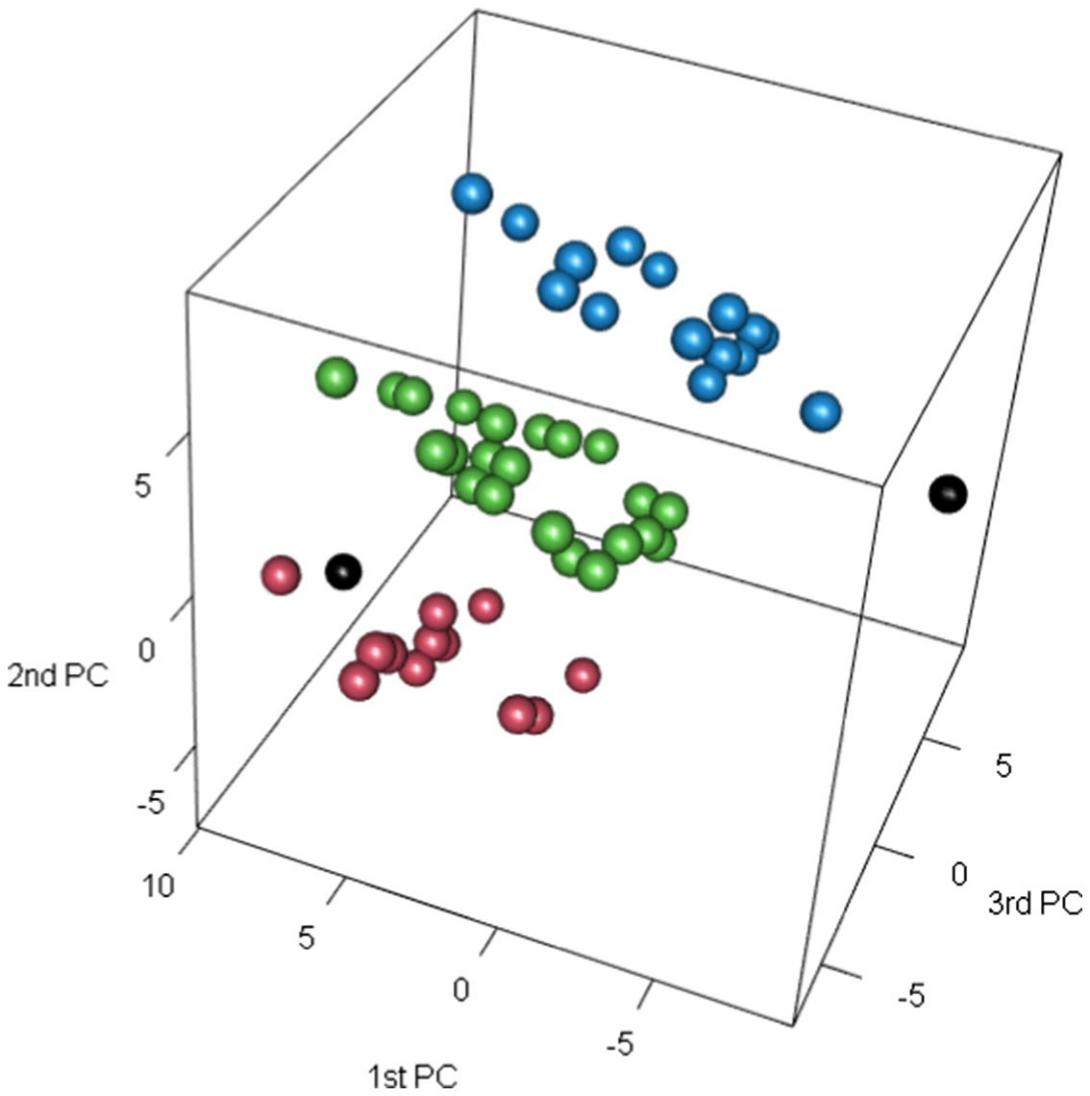
Respondents scores (with groupings colored and single group combinations in black) on three extracted PCs (from preferences of alternative wine communication).

After plotting the respondents scores on the three extracted PCs, see Figure 1, we identified three groups (plus two deviant single combination groups) of observations which (by construction) differed significantly in terms of their preferences of alternative wine communication, see Table 6.

The number of preferences also differed significantly between the groups, the bottom group (in red, named G1) with fewest (0–3), the middle group (green, G2) with slightly more (1–4) and the top group (blue, G3) with most preferences (2–5), see Figure 1. and Table 7. The left single combination group (black, G4) had 80 respondents preferring only Shapes/colors, while the utmost right (black, G5) had four respondents preferring all the seven ways of alternative wine communication.

**Table 7:** General characteristics and preferences for wine communication by group (G1-G5).

General characteristics					Preference for altern. wine communication (%)						
Group	Nr of respondents***	Average nr of alternatives for communication**	Average age (years)*	Median income (1,000 SEK)	Shapes/colors (%)***	Sounds (%)***	Music (%)***	Speech (%)***	Touch (%)***	Odors/aromas (%)***	Flavors (%)***
G1	57	1.04	37.6	37.4	33	9	14	21	26	0	0
G2	111	1.72	40.5	37.0	37	6	7	7	14	40	60
G3	77	2.83	43.5	38.0	44	5	8	8	18	100	100
G4	80	1	43.5	41.0	100	0	0	0	0	0	0
G5	4	7	33.0	24.5	100	100	100	100	100	100	100

Test of equal size: Nr of respondents (Chi-square). Test of association: Preference for alternative (Chi-square). Test of equal location: Age (Anova F-test); Nr of alternatives and Income (Kruskal-Wallis). *p*-value: 0.05 > * > 0.01 > ** > 0.001 > ***.

Relatively many in group G1 preferred hearing and tactile, but fewer in G2 and G3. While none in group G1 preferred smells and taste, about half in G2 and everyone in G3 did. The groups also differed significantly in terms of age (G5 youngest, G3 and G4 oldest), see Table 7, and to the degree which they selected wine with regard to sustainability (Table AG.1).

In studying the relation to purchase choice and consumption experience we use multinomial regression and regress the group (but to enable reliable estimation we exclude group G5) on the locale (as to rule it its effect) and the influential factor under study, see Table AG.2. In addition to previously found significant factors (see sections 3.2.1–7) influencing purchase (country of origin, grape, illustrations on the label, vintage), the wine producer was also found to be significant. Regarding consumption experience (Table AG.3) the situation was similar (with smell/odor and tactile sensations, overall room environment and hosts/staffs from before) adding visual impression.

We summarize the description of the groups in Table 8.

**Table 8:** Salient features among groups compared to the least salient (reference) group.

Group (size in %)	Alt. wine communication	Demographics and other characteristics	Factors influencing purchase choice	Factors influencing consumption experience
G1 (17%)	Hearing, tactile	Younger, consume in bars/restaurants, willing to try blended wine	Country of origin, illustrations on the label, wine producer	Tactile sensations
G2 (34%)		Consume with friends/family/wine tasting group, regard sustainability	Grape	Overall room environment, hosts/professionals/ staff
G3 (23%)	Smell, taste	Older, consume at home, consider buying with only sensory information	Country of origin, wine producer, vintage	Visual impression, smell/odor sensation
G4 (24%)	Visual	Older, consume with friends/family/wine tasting group, regard sustainability		Hosts/professionals/staff

### 3.4. Most frequent combinations of reported alternatives to regular communication of wine

Given the frequencies in Table 3 it is as expected that the most frequent combinations (63.2%) of reported alternatives (see Table 9) only involved the univariately most frequent alternatives: shapes/colors (54.1%), flavors (38.0%) and odors/aromas (45.0%). Almost half of those who reported any three alternatives (13.7%) specifically reported these three (6.4%). However, no one reported the specific combination of shapes/colors and odors/aromas. The most common combination was the group G4 (see section 3.3) with 80 respondents only preferring shapes/colors as alternative communication.

**Table 9:** Observed combinations of the most frequently reported alternatives to regular wine communication (shapes/colors, flavors and odors/aromas).

Shapes/Colors (other visual symbols)	Flavors (tastes)	Odors/aromas (smells)	Sound; Music; Speech; Touch	*n*	%
YES	NO	NO	NO	80	24.3
NO	YES	NO	NO	37	11.2
NO	YES	YES	NO	35	10.6
YES	YES	YES	NO	21	6.4
YES	YES	NO	NO	18	5.5
NO	NO	YES	NO	17	5.2
YES	NO	YES	NO	0	0
Sum of observations among the combinations in the table				208	63.2
All observations				329	100

It is hard to detect significant differences between the combinations since they are relatively small (except for G4). Still, the age of respondents was significantly higher among the combination odors/aromas and flavors (49.7 years), relative to those only reporting the alternative odors/aromas (37.9 years), but not among those only preferring flavors (45.4 years), see Figure AH.1. A few influential factors on the choice of purchase (illustrations on the label, sensory indicators), see Table AH.1, and on the consumption experience (taste sensations), see Table AH.2, differed significantly between the combinations. Due to scarcity of observations, we did not control for the locale.

## 4. Discussion

The findings of this study revealed that taste (94%), tasting/dinner setting (66%), and the smell of the wine (65%) were the most influential self-reported sensory factors during wine consumption. In contrast, a smaller percentage of participants reported being influenced by other senses, such as vision (26%), touch (12%), and sound (8%). These results suggest that there is relatively low awareness of crossmodal correspondence and the effects of the multisensory environment among this group of engaged wine consumers. This highlights the potential for more serious attempts to implement the findings of this research in the food industry, considering the valuable role that alternative multisensory communication tools have played in sensory marketing and increasing sales (Elder and Krishna, 2010; Varela and Ares, 2012; Spence et al., 2013; Krishna and Schwarz, 2014; Croijmans and Wang, 2021; Spence, 2022a).

Despite the extensive research demonstrating the influence of senses like vision and touch on crossmodal correspondence (Lick et al., 2017; Baptista et al., 2022; Nguyen and Durner, 2023; Spence, 2023) and the role of haptic sensations (Piqueras-Fiszman and Spence, 2012; Wang and Spence, 2018), the participants in this study reported these factors as being influential for only a few of them. However, when asked if they would consider purchasing wine based solely on sensory information, the majority of participants reacted positively, particularly if the sensory descriptors aligned with their personal preferences (91%). Similarly, when asked about purchasing a wine with no specific origin but blended from different wines, participants responded positively (95%) if the descriptors matched their sensory profile.

Furthermore, when it came to the primary choice of alternative communication for wine, visual cues were rated the highest (54%). This supports the idea that this group of participants is open to buying wine based solely on sensory information, without considering origin or blending. By analyzing the respondents’ scores on the three extracted principal components, three groups (along with two deviant single combination groups) were identified. These groups exhibited significant differences in their preferences for alternative wine communication. This result suggests the potential for a strategic communication approach that employs different forms of communication tools to target different consumer groups through alternative communication methods. It also highlights the importance of utilizing such tools to support specific consumer groups in need of special assistance (Spence, 2022c).

As mentioned earlier, one of the motivations behind this research is to explore how novel sustainable products that are resource-efficient and have a reduced carbon footprint can be effectively communicated and validated for consumer acceptance (Lévy et al., 2006; Peschel et al., 2019; Herdenstam et al., 2022). This research aims to enhance researchers’ understanding of how consumers perceive a product in relation to meeting their expectations, thereby contributing to the communication and acceptance of such products.

## 5. Conclusion

These results provide insights into the factors that influence wine purchasing and consumption, as well as the preferences and attitudes towards alternative wine communication. They highlight the importance of sensory information, particularly taste, and the potential for using visual and other alternative means to communicate the sensory experience of wine. Understanding these preferences and associations can assist in developing effective strategies for wine marketing and communication, addressing better resource use. Overall, these findings suggest that wine consumers consider various factors when purchasing and consuming wine, including sensory indicators, personal preferences, and influential factors. There is openness to alternative means of wine communication, particularly visual communication using shapes and colors. The associations between preferences and demographics/influential factors highlight the individual differences in wine communication preferences. It’s important to note that these conclusions are based on the information provided in the given sections. Further analysis and research may be necessary to validate and expand upon these findings.

## Data availability statement

The original contributions presented in the study are included in the article/Supplementary materials, further inquiries can be directed to the corresponding author.

## Ethics statement

Ethical review and approval was not required for the study on human participants in accordance with the local legislation and institutional requirements. Written informed consent was taken from the participants.

## Author contributions

AC-F contributed to conception and design of the study and wrote the first draft of the manuscript. CS wrote sections of the manuscript and approved the submitted version. AC-F and CS contributed to manuscript revision and read. NP performed the statistical analysis and contributed to manuscript revision. All authors contributed to the article and approved the submitted version.

## Conflict of interest

The authors declare that the research was conducted in the absence of any commercial or financial relationships that could be construed as a potential conflict of interest.

## Publisher’s note

All claims expressed in this article are solely those of the authors and do not necessarily represent those of their affiliated organizations, or those of the publisher, the editors and the reviewers. Any product that may be evaluated in this article, or claim that may be made by its manufacturer, is not guaranteed or endorsed by the publisher.
